# Type‐III Superconductivity

**DOI:** 10.1002/advs.202206523

**Published:** 2023-03-25

**Authors:** M. Cristina Diamantini, Carlo A. Trugenberger, Sheng‐Zong Chen, Yu‐Jung Lu, Chi‐Te Liang, Valerii M. Vinokur

**Affiliations:** ^1^ NiPS Laboratory INFN and Dipartimento di Fisica e Geologia University of Perugia via A. Pascoli Perugia I‐06100 Italy; ^2^ SwissScientific Technologies SA rue du Rhone 59 Geneva CH‐1204 Switzerland; ^3^ Department of Physics National Taiwan University Taipei 106 Taiwan; ^4^ Research Center for Applied Sciences Academia Sinica Taipei 115 Taiwan; ^5^ Center for Quantum Science and Engineering National Taiwan University Taipei 106 Taiwan; ^6^ Terra Quantum AG Kornhausstrasse 25 St. Gallen CH‐9000 Switzerland

**Keywords:** Berezinskii–Kosterlitz–Thouless transition, superconductivity, vortex deconfinement

## Abstract

Superconductivity remains one of most fascinating quantum phenomena existing on a macroscopic scale. Its rich phenomenology is usually described by the Ginzburg–Landau (GL) theory in terms of the order parameter, representing the macroscopic wave function of the superconducting condensate. The GL theory addresses one of the prime superconducting properties, screening of the electromagnetic field because it becomes massive within a superconductor, the famous Anderson–Higgs mechanism. Here the authors describe another widely‐spread type of superconductivity where the Anderson–Higgs mechanism does not work and must be replaced by the Deser–Jackiw–Templeton topological mass generation and, correspondingly, the GL effective field theory must be replaced by an effective topological gauge theory. These superconductors are inherently inhomogeneous granular superconductors, where electronic granularity is either fundamental or emerging. It is shown that the corresponding superconducting transition is a 3D generalization of the 2D Berezinskii–Kosterlitz–Thouless vortex binding–unbinding transition. The binding–unbinding of the line‐like vortices in 3D results in the Vogel‐Fulcher‐Tamman scaling of the resistance near the superconducting transition. The authors report experimental data fully confirming the VFT behavior of the resistance.

## Graphical abstract

The effective field theory of inhomogeneous superconductors is not the Ginzburg–Landau theory but a topological gauge theory. Vortices with no dissipative core can proliferate also with no magnetic field. When the temperature is high enough, vortex deconfinement destroys superconductivity, generalizing the Berezinskii–Kosterlitz–Thouless transition mechanism to any dimension.

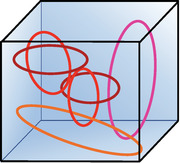

## 1 Introduction

The macroscopic physics of traditional superconductors (SC) is governed by the Ginzburg–Landau (GL) model, see for example, ref. [1], describing superconductors in terms of a local order parameter. The ground state of a macroscopic superconductor of a size much exceeding the London penetration depth, $\lambda_{L}$, has an order parameter that is constant in the system's bulk, outside a boundary shell of the width $\lambda_{L}$. There are, however, superconductors, for example thin films of a thickness *d* comparable with the coherence length ξ, which are characterized by a completely different ground state exhibiting a paradigmatic granularity.^[^ ^2^, ^3^ ^]^ In these systems superconductivity sets in when global phase coherence is established due to tunneling percolation of the Cooper pairs between droplets of locally formed condensate. The granularity of these systems is associated with a superconductor‐to‐superinsulator quantum phase transition^[^ ^4^, ^5^, ^6^ ^]^ which may occur via an intermediate Bose metal^[^ ^4^, ^7^, ^8^ ^]^ phase, see refs. [9, 10, 11], and has been detected experimentally.^[^ ^12^, ^13^ ^]^

Two important features characterize these planar “self‐granular” superconductors. First, the gauge screening length, the Pearl length $\lambda_{P}=\lambda_{L}^{2}/d$ due to the familiar Anderson‐Higgs mechanism, see e.g. [1], would become larger than the experimental system size for small *d*. Second, near the quantum transition, the electric fields induced by charges residing in the system remain in‐plane over the whole sample because of the large dielectric constant.^[^ ^9^, ^10^, ^11^ ^]^ Usually, such electric fields are not very relevant in standard superconductors. In our planar superconductors, however, these 2D electric fields are much stronger than the usual 3D ones, since they decay as 1/*r* with the increasing distance *r* from the charge and cannot be neglected. When coupled to electromagnetism, the time‐dependent Ginzburg–Landau model becomes, therefore (non‐relativistic) scalar quantum electrodynamics (QED), which is ill defined in 2D because of its infrared divergences tied to the perturbative coupling scaling as ln(*L*/ξ), where *L* is the sample size.^[^ ^14^, ^15^ ^]^ If one tries to derive the free energy for a putative order parameter from the elastic interaction with a substrate one obtains a non‐local functional describing a self‐organized array of superconducting islands,^[^ ^16^ ^]^ confirming that the original GL model breaks down. Hence, for planar systems with long‐range interactions, the local Ginzburg–Landau model does not provide an adequate description of global superconductivity^[^ ^17^ ^]^ and can only address local superconductivity within droplets of the typical size of order ξ.

Notably, emergent granularity is not confined to thin films. Recently, the same physics has been detected in bulk samples.^[^ ^18^ ^]^ Even more importantly, the ground‐state of the high‐*T*_c_ cuprates is inhomogeneous, especially in the underdoped regime and the same percolation model is thought to be responsible for global superconductivity.^[^ ^19^, ^20^ ^]^ In this type of percolating superconductivity, electron pairs survive above *T*_c_, which is the case both in 2D^[^ ^21^ ^]^ and 3D,^[^ ^22^ ^]^ and there is a quantum transition to an insulating state, both in 2D, see refs. [9, 10, 11], and 3D.^[^ ^23^ ^]^ In 2D, the fragmentation into separate condensate droplets is due to strong infrared divergences near the quantum insulating transition.^[^ ^17^ ^]^ In general, however, this type of behavior seems characteristic of superconductors in which the pairing mechanism is not the BCS one^[^ ^1^ ^]^ but arises from a stronger attractive interaction leading to pronounced bosonic pairs of a size much smaller than their typical separation distance and forming a Bose–Einstein condensate (BEC). The BECs with long‐range dipole interactions of particles carrying magnetic moments are known to fragment into liquid droplets due to strong quantum fluctuations.^[^ ^24^ ^]^

Here we formulate the effective long‐distance gauge theory of inhomogeneous superconductors in 3D and show that superconductivity sets in via the Vogel–Fulcher–Tammann (VFT) transition, the 3D counterpart of the 2D Berezinskii–Kosterlitz–Thouless (BKT) topological phase transition, and we report an experiment confirming the VFT scaling of the resistance at the transition. The crucial point is that, contrary to type II superconductors, vortices in inhomogeneous superconductors are not Abrikosov vortices but, rather, mobile vortices with no dissipative core which arise due to non‐trivial phase circulations on adjacent droplets. Charges on the droplets and vortices between them have unavoidable topological mutual statistics interactions and a local descriptions of these requires the introduction of gauge fields.^[^ ^25^ ^]^ Therefore, the effective field theory for these inhomogeneous superconductors must be a gauge theory. We refer to this novel, topologically driven superconducting state (in any dimension) as to type‐III superconductivity. This choice is dictated by the standard classification of superconductors with respect to penetration of the applied magnetic field. Type I superconductors expel magnetic field *H* and are referred to be in a Meissner state at *H* < *H*_c_, while at the critical field *H*_c_ superconductivity is destroyed. In type II superconductivity *H*_c_ “splits” into the lower, *H*_c1_, and upper, *H*_c2_, critical fields. At *H* < *H*_c1_ type II superconductors are in the Meissner state; at *H*_c1_ < *H* < *H*_c2_ (mixed or vortex state) a magnetic field penetrates type II superconductors in form of Abrikosov vortices having a normal core; at *H* = *H*_c2_ vortex normal cores overlap and superconductivity gets destroyed. In type III superconductors *H*_c1_ = 0 and vortices, which, as mentioned above, in this case do not possess a normal core, can penetrate at any magnetic field (corresponding to a flux at least equal to a quantum flux), and there is no true Meissner state. Superconductivity is not destroyed at low temperatures because quantum diffusion of vortices is suppressed by a large corresponding term in the action. This behavior has been experimentally detected^[^ ^26^ ^]^ in Josephson junction arrays, the paradigmatic example of type III superconductors in 2D. The response can be both diamagnetic and paramagnetic; however, since it is preferentially paramagnetic, this state is often called paramagnetic Meissner state. Finally, in type III superconductors, vortices can proliferate even without a magnetic field when the temperature is high enough.

## 2 BKT and VFT Transitions

As we have established in Section 1, the local GL model fails to provide a consistent description of granular or droplet‐composed superconductors^[^ ^20^ ^]^ and has thus to be replaced by a generalized gauge theory introduced in refs. [27, 28] and recently discussed in detail in 2D in ref. [17]. One of the fundamental implications of the gauge theory of granular superconductors is that its vortices, contrary to Abrikosov vortices, have no dissipative core, since they arise from non‐trivial circulations of the local phases of the condensate on adjacent droplets. Hence, superconductivity in these materials that do not possess a global order parameter is referred to as “Higgsless superconductivity.”^[^ ^28^ ^]^ Furthermore, since the ground state of Higgsless superconductors may carry topological order, they are also called “superconductors with topological order.”^[^ ^27^ ^]^

Since superconductivity is realized by global phase coherence being established over all pre‐existing condensate droplets, its destruction is caused by a proliferation of vortices and not by breaking of Cooper pairs, as in traditional superconductors. In 2D, this is the famed BKT transition, see ref. [29] for a review, resulting in the BKT resistance scaling

(1) $$R\left(T\right)\propto e^{-\sqrt{\frac{b}{|T-T_{\text{BKT}}|}}}\;$$

where *b* is a constant having the dimensionality of temperature. In 3D, the phase transition is again caused by vortex liberation, but the vortices are now 1D extended objects, magnetic strings. The superconducting phase transition is thus caused by 1D strings becoming tensionless. This transition has been studied in ref. [30]. The corresponding behavior of the resistance is modified to the VFT scaling

(2) $$R\left(T\right)\propto e^{-\frac{b^{\prime }}{|T-T_{\text{VFT}}|}}\;$$

This same dual scaling for vanishing conductivity and due to electric strings becoming tensionless, see ref. [11] for a review, has already been detected at the superinsulating side of the quantum transition ref. [31] and has also been obtained in the XY model with quenched disorder, which apparently is equivalent to one more effective space dimension.^[^ ^32^, ^33^ ^]^

## 3 Gauge Theory of Type‐III Superconductors

We model an inhomogeneous superconductor by a cubic lattice, with the sites representing the droplets and the links encoding possible tunneling junctions between them. The fundamental degrees of freedom of the model are of two types. First, there are integer (in units of 2*e*) charges *Q*_0_ located at the sites and currents *Q*_*i*_ on the links of the lattice. Together they constitute a four‐current *Q*_μ_, with Greek letters standing for the space‐time indices. In the absence of background charges, this current is conserved, *d*_μ_ *Q*_μ_ = 0 with *d*_μ_ denoting the forward lattice derivative in the direction μ; summation over equal Greek indices is implied. Conservation requires that only closed loops *Q*_μ_ are allowed on the lattice, representing charge‐hole fluctuations. The second type of excitations are integer (in units 2π/2*e*, we use natural units *c* = 1, ℏ = 1, ε_0_ = 1) coreless Josephson vortices that arise from the nontrivial circulations of the local condensate phases on the droplets. Since these circulations are 1D extended objects they are represented by integer lattice plaquette variables *M*_μν_. Since the vortices that we consider are closed loops, these are also conserved, *d*_µ_ *M*_μν_ = *d*_ν_ *M*_μν_ = 0 and describe, thus, closed surfaces representing the nucleation and subsequent annihilation of a vortex loop. Open vortices with magnetic monopoles at their ends are also possible^[^ ^34^ ^]^ but are not relevant for what follows.

The infrared (IR) dominant interaction between charges and vortices is topological, it encodes their mutual statistical interaction, that is, the Aharonov–Bohm^[^ ^35^ ^]^ and Aharonov–Casher^[^ ^36^ ^]^ (ABC) phases accumulating when one type of excitation encircles the other. In the Euclidean partition function, which we will consider in the rest of this paper, the ABC topological interactions are accounted for by an imaginary term representing the Gaussian linking of the closed loops and surfaces in four Euclidean dimensions.^[^ ^37^ ^]^ As pointed out by Wilczek,^[^ ^25^ ^]^ this interaction can also be represented in local form by introducing two fictitious gauge fields interacting with the two types of excitations and with a topological coupling to each other. In 2D this is the well known Chern–Simons term;^[^ ^38^ ^]^ in the 3D case, this is the three‐index BF term,^[^ ^11^ ^]^ ϵ_μναβ_∂_ν_, coupling an effective gauge field *a*_μ_ for the charges with the two‐index effective gauge field *b*_αβ_ for the 1D extended vortices. Here ϵ^μναβ^ is the usual totally antisymmetric tensor. Then the Euclidean action acquires the form

(3) $$S=\sum_{x}i\frac{\ell^{4}}{4\pi }a_{\mu }k_{\mu \alpha \beta }b_{\alpha \beta }+i\ell a_{\mu }Q_{\mu }+i\ell^{2}\frac{1}{2}b_{\mu \nu }M_{\mu \nu }\;$$

where *x* denotes the lattice sites, ℓ is the link length, and *k*_μαβ_ is the lattice BF term, described in detail below.

The two gauge fields are invariant under the gauge transformations

(4) $$\begin{array}{ccc} a_{\mu } & \to & a_{\mu }+d_{\mu }\xi \;\\ b_{\mu \nu } & \to & b_{\mu \nu }+d_{\mu }\lambda_{\nu }-d_{\nu }\lambda_{\mu }\; \end{array}$$

reflecting the fact that the charge world‐lines and vortex world‐surfaces they couple to are closed. Note that, at the classical level, the equations of motion imply that the gauge fields themselves encode the charge and vortex currents, respectively,

(5) $$\begin{array}{c} Q_{\mu }=-\frac{1}{4\pi }\ell^{3}k_{\mu \alpha \beta }b_{\alpha \beta }\;\\ M_{\mu \nu }=-\frac{1}{2\pi }\ell^{2}k_{\mu \nu \alpha }a_{\alpha }\; \end{array}$$

If *a*_μ_ is a vector field and *b*_μν_ a pseudotensor field, the model is also invariant under the parity P and time‐reversal T symmetries. In general, the BF action for a model defined on a compact space with non‐trivial topology has a ground state degeneracy^[^ ^40^ ^]^ reflecting the topology, exactly as the Chern–Simons term in 2D. However, when the coefficient of the BF term is 1/4π, as in Equation (3), the ground state is unique.^[^ ^40^ ^]^

Having established that the effective field theory for the inhomogeneous superconductors is a generalized gauge theory we can proceed as usual in its construction, adding order by order all interactions that respect the relevant symmetries. In this case, the next‐order gauge‐invariant terms are the kinetic terms for the two gauge fields. For the vector gauge field *a*_μ_ this is the usual Maxwell term, constructed from the field strength *f*_μν_ = *d*_μ_ *a*_ν_ − *d*_ν_ *a*_μ_. For the antisymmetric tensor gauge field *b*_μν_, the kinetic term is quadratic in the field strength

(6) $$h_{\mu \nu \alpha }=d_{\mu }b_{\nu \alpha }+d_{\nu }b_{\alpha \mu }+d_{\alpha }b_{\mu \nu }\;$$

It is easy to check that this is invariant under a gauge transformation (Equation (4)). Adding these next‐order terms, we obtain the Euclidean effective action

(7) $$\begin{array}{ccc} S & = & \sum_{x}\frac{\ell^{4}}{4f^{2}}f_{\mu \nu }f_{\mu \nu }+i\frac{\ell^{4}}{4\pi }a_{\mu }k_{\mu \alpha \beta }b_{\alpha \beta }+\frac{\ell^{4}}{12\Lambda^{2}}h_{\mu \nu \alpha }h_{\mu \nu \alpha }\\ & & +\;i\ell a_{\mu }Q_{\mu }+i\ell^{2}\frac{1}{2}b_{\mu \nu }M_{\mu \nu } \end{array}$$

The dimensionless parameter f=O(e) represents the effective Coulomb interaction strength in the material and 1/Λ is the magnetic screening length. The two dimensionless parameters *f* and Λℓ encode the strengths of the electric and magnetic interactions, respectively. Non‐relativistic effects can be easily incorporated by considering a velocity of light *v* < 1 but are of no particular relevance for what follows.

We now integrate out the emergent gauge fields to obtain an effective (Euclidean) action for the charges and vortices alone,

(8) $$\begin{array}{ccc} S_{\mathrm{QM}} & = & \sum_{x}\frac{f^{2}}{2\ell^{2}}\;Q_{\mu }\frac{\delta_{\mu \nu }}{m^{2}-\nabla^{2}}Q_{\nu }+\frac{\Lambda^{2}}{8}\;M_{\mu \nu }\frac{\delta_{\mu \alpha }\delta_{\nu \beta }-\delta_{\mu \beta }\delta_{\nu \alpha }}{m^{2}-\nabla^{2}}M_{\alpha \beta }\\ & & +\;i\frac{\pi m^{2}}{\ell }Q_{\mu }\frac{k_{\mu \alpha \beta }}{\nabla^{2}\left(m^{2}-\nabla^{2}\right)}M_{\alpha \beta }\; \end{array}$$

where

(9) $$m=\frac{f\Lambda }{2\pi }\;,$$

is the gauge‐invariant, topological mass, analogous to the famed Chern–Simons mass in 2D.^[^ ^38^, ^39^ ^]^ This is one of the main points of this paper: the topological mutual statistics interaction screens both the vortex–vortex interaction and the Coulomb interaction between charges. Approximating these screened potentials by delta functions one can estimate the mass (coefficient multiplying the world‐line length in the action) of charges and the string tension (coefficient multiplying the world‐sheet area in the action) of vortices. In this phase of the system both charges and vortices are gapped excitations with mass *M* = *f* ^2^/(2*m* ^2^ℓ^3^) and string tension *T* = Λ^2^/(8*m* ^2^ℓ^2^) interacting via short‐range screened potentials. For temperatures low enough this is thus a thermally activated, insulating phase, for higher temperatures it is a metal.

Let us now investigate what happens when charges condense, which can be described by letting the original integer‐valued variable *Q*_μ_ become a real‐valued field over which one has to integrate (as opposed to sum) in the partition function. Formally, this amounts to using the Poisson summation formula

(10) $$\sum_{\left\{Q_{\mu }\right\}}f\left(Q_{\mu }\right)=\sum_{\left\{k_{\mu }\right\}}\int dQ_{\mu }f\left(Q_{\mu }\right)e^{i2\pi Q_{\mu }k_{\mu }}\;$$

and focusing only on the sector in which the dual variable *k*_μ_ = 0. However, since the current *Q*_μ_ is conserved and thus constrained by the equation *d*_μ_ *Q*_μ_ = 0, we must first introduce the representation *Q*_μ_ = ℓ*k*_μαβ_ *n*_αβ_. The new variables *n*_αβ_ are now free but redundant, since they can be gauge transformed as in Equation (4). There are only three gauge‐invariant degrees of freedom in the *n*_αβ_, corresponding to the three unconstrained variables *Q*_μ_. The removal of the three redundant variables can be taken care of by the usual gauge fixing in the integration.

To this end we consider a 4D Euclidean lattice with spacing ℓ representing the typical distance of the superconducting droplets. Let *d*_μ_, ${\hat{d}}_{\mu }$, *S*_μ_, and ${\hat{S}}_{\mu }$ denote forward and backward lattice derivatives and shifts. Then the forward and backward lattice BF terms are defined by the three‐index operators^[^ ^4^ ^]^

(11) $$\begin{array}{c} k_{\mu \nu \rho }\equiv S_{\mu }\varepsilon_{\mu \alpha \nu \rho }d_{\alpha }\\ {\hat{k}}_{\mu \nu \rho }\equiv \varepsilon_{\mu \nu \alpha \rho }{\hat{d}}_{\alpha }{\hat{S}}_{\rho } \end{array}$$

where no summation over equal indices μ and ρ is implied. The two lattice BF operators are interchanged (no minus sign) upon summation by parts on the lattice and are gauge invariant,

(12) $$\begin{array}{c} k_{\mu \nu \rho }d_{\nu }=k_{\mu \nu \rho }d_{\rho }={\hat{d}}_{\mu }k_{\mu \nu \rho }=0\;\\ {\hat{k}}_{\mu \nu \rho }d_{\rho }={\hat{d}}_{\mu }{\hat{k}}_{\mu \nu \rho }={\hat{d}}_{\nu }{\hat{k}}_{\mu \nu \rho }=0\; \end{array}$$

They also satisfy the identities

(13) $$\begin{array}{ccc} {\hat{k}}_{\mu \nu \rho }k_{\rho \lambda \omega } & = & -\left(\delta_{\mu \lambda }\delta_{\nu \omega }-\delta_{\mu \omega }\delta_{\nu \lambda }\right)\nabla^{2}+\left(\delta_{\mu \lambda }d_{\nu }{\hat{d}}_{\omega }-\delta_{\nu \lambda }d_{\mu }{\hat{d}}_{\omega }\right)\\ & & +\;\left(\delta_{\nu \omega }d_{\mu }{\hat{d}}_{\lambda }-\delta_{\mu \omega }d_{\nu }{\hat{d}}_{\lambda }\right)\;\\ {\hat{k}}_{\mu \nu \rho }k_{\rho \nu \omega } & = & k_{\mu \nu \rho }{\hat{k}}_{\rho \nu \omega }=2\left(\delta_{\mu \omega }\nabla^{2}-d_{\mu }{\hat{d}}_{\omega }\right)\; \end{array}$$

where $\nabla^{2}={\hat{d}}_{\mu }d_{\mu }$ is the lattice Laplacian.

Using Equation (13) we write the action Equation (8) as

(14) $$\begin{array}{ccc} S_{\mathrm{nM}} & = & \sum_{x}\left[\frac{\Lambda^{2}}{8}\;M_{\mu \nu }\frac{\delta_{\mu \alpha }\delta_{\nu \beta }-\delta_{\mu \beta }\delta_{\nu \alpha }}{m^{2}-\nabla^{2}}M_{\alpha \beta }\right.\\ & & +\;\frac{f^{2}}{2}n_{\mu \nu }\frac{-\left(\delta_{\mu \alpha }\delta_{\nu \beta }-\delta_{\mu \beta }\delta_{\nu \alpha }\right)\nabla^{2}+\left(\delta_{\mu \alpha }d_{\nu }{\hat{d}}_{\beta }-\delta_{\nu \alpha }d_{\mu }{\hat{d}}_{\beta }\right)}{m^{2}-\nabla^{2}}n_{\alpha \beta }\\ & & +\;\frac{f^{2}}{2}n_{\mu \nu }\frac{\delta_{\nu \beta }d_{\mu }{\hat{d}}_{\alpha }-\delta_{\mu \beta }d_{\nu }{\hat{d}}_{\alpha }}{m^{2}-\nabla^{2}}n_{\alpha \beta }\\ & & \left.-\;i\pi m^{2}n_{\mu \nu }\frac{\delta_{\mu \alpha }\delta_{\nu \beta }-\delta_{\mu \beta }\delta_{\nu \alpha }}{m^{2}-\nabla^{2}}M_{\alpha \beta }\right]\; \end{array}$$

Performing Gaussian integration over the field *n*_μν_, we obtain

(15) $$S_{M}=\sum_{x}\frac{\Lambda^{2}}{8}\;M_{\mu \nu }\frac{\delta_{\mu \alpha }\delta_{\nu \beta }-\delta_{\mu \beta }\delta_{\nu \alpha }}{-\nabla^{2}}M_{\alpha \beta }\;$$

This is the second main result of this paper. The global condensation in the superconducting phase turns the vortex interaction into a long‐range one, suppressing the topological screening. As has been derived in ref. [4], a 4D Coulomb potential for the elements of the world‐surface of a vortex implies that the self‐energy of a circular vortex‐loop of radius *r* scales like *r* ln*r*, which amounts to logarithmic vortex confinement as is the case in 2D. In these inhomogeneous 3D superconductors, thus, the destruction of global superconductivity via tunnelling percolation also takes place by vortex liberation, similarly to the 2D BKT transition. Since vortices are 1D objects, this happens when the effective string tension of the vortices, including the entropy correction, vanishes. This transition has been studied in ref. [30] and leads to the VFT critical behavior of the resistance given by Equation (2).

## 4 Experiment

**Figure 1:**
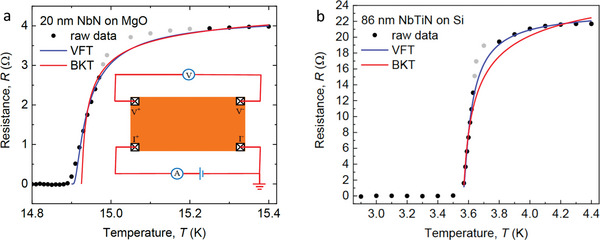
Resistance measurements. a) Four‐terminal dc resistance measurements of the 20‐nm‐thick NbN film at different temperatures. The red and blue curves correspond to BKT fitting and VFT fitting to the experimental data, respectively. The gray points mark experimental data deviating from the fits. The inset depicts a sketch of the four‐terminal dc resistance measurements of NbN and NbTiN films. Electrodes V^+^ and V^−^ correspond to the two voltage probes for measuring the voltage difference. Electrodes I^+^ and I^−^ are the source and drain contacts. The symbols V and I stand for the voltmeter and ammeter measuring the voltage difference and current, respectively. b) Four‐terminal dc resistance measurements of a 20‐nm‐thick NbN film at different temperatures. The red and blue curves correspond to the BKT fitting and VFT fitting to the experimental data, respectively. The three data points marked gray show the noticeable deviation from the fits.

Standard four‐terminal dc resistance measurements, see the inset in **Figure** **1**a, are taken on films of the nitrides of the transition metals, NbTiN and NbN. The detailed preparation of the 20‐nm‐thick NbN film on an MgO substrate can be found in Experimental Section. The zero‐temperature coherence length of this film is measured to be (4.40 ± 0.05) nm, that is, much shorter than the film thickness *d* = 20 nm  hence the NbN film is a 3D superconductor. The details of the preparation of the disordered nonstoichiometric 86‐nm‐thick NbTiN film can be found in ref. [41]. This film is deposited on a Si (100) substrate by radio frequency (RF) reactive magnetron co‐sputtering from two separate NbN and TiN targets. As the film is deposited on a Si substrate, it is fully compatible with the existing Si CMOS technology. We benefit from the fact that this NbTiN film has been studied before.^[^ ^41^ ^]^ The zero‐temperature coherence length is measured to be (9.53  ±  0.04) nm, which is much shorter than the film thickness 86 nm.^[^ ^41^ ^]^ Therefore, we also have a 3D superconductor system. Both samples are approximately rectangular films with the length of ≈ 4.5 mm and the width of ≈ 3.5 mm. As shown later, we see that the VFT model fits the data much better than the corresponding BKT one does.

Figure 1a shows the four‐terminal dc resistance measurements of the 20‐nm‐thick NbN film grown on the MgO substrate as a function of temperature *T*. We observe a rather broad metal‐superconductor transition with decreasing *T*. In order to further study this, we fit our experimental results with the BKT‐(red curve) and the VFT‐(blue curve) scaling resistivity. Note that we use the same three fitting parameters, the critical temperature, the overall normalization of the resistance, and a constant *b* having the dimensionality of temperature for the two fits. The aim is to compare the two scalings and identify the best one. Marking the six data points that significantly deviate from any fit in gray, we see that the VFT dependence fits the experimental results much better than the BKT one does, see Figure 1a. Analogous study is performed on the much thicker NbTiN film with a thickness of 86 nm deposited on a Si substrate. Figure 1b displays the same kind of the four‐terminal dc resistance measurements. Again, we observe a broad metal‐superconductor transition with decreasing temperature. The BKT scaling clearly fails to fit the experimental data. The VFT fits describe the experimental data very well, except for three points marked gray. While at present no concrete model for this deviation, which could be caused by nonstoichiometric disorder, can be offered, one can strongly assume that it is caused by quantum corrections to the resistance which become essential in the close vicinity of the superconducting transition temperature *T*_c_ which noticeably exceeds *T*_VFT_. The fact that in thicker (86 nm thick) NbTiN film these deviations are much smaller than in the 20 nm thick NbN film excellently agrees with this assumption. However, detailed calculations beyond the scope of the present work are necessary to quantitatively explore this assumption.

**Figure 2:**
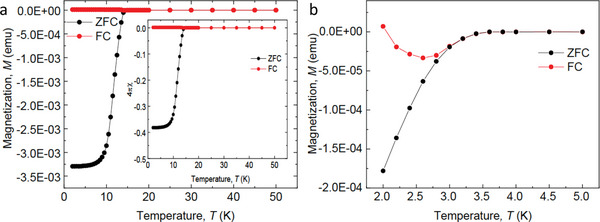
Magnetization measurements under zero‐field‐cooled (ZFC) and field‐cooled (FC) condictions. a) Magnetization of the 20 nm‐thick NbN film. Inset: magnetic susceptibility. The measurements are taken under the external field of 20 Oe. b) Magnetization of the 86 nm‐thick NbTiN film. The measurements are taken under the external field of 5 Oe.

The next step is to confirm that the observed resistance behavior reflects the genuine bulk superconducting properties rather than stems from the local superconductivity that might arise at the surface or 1D defect filaments of the investigated samples. To that end, we perform magnetic susceptibility measurements. As shown in **Figure** **2**, the investigated systems demonstrate strong diamagnetic response in the magnetic susceptibility, evidencing that the observed superconductivity is the genuine bulk superconductivity for both films. The downward and then upward behavior of the magnetic susceptibility in the 86‐nm‐thick NbTiN film in the FC condition indicates possible paramagnetic Meissner effect (PME).^[^ ^42^ ^]^ The PME generally appears in superconductors with strong vortex pinning at low temperatures.

## 5 Conclusion

Our results reveal the existence of a novel type of superconductivity, which we call type‐III superconductivity. The standard superconductivity is well described by the local Ginzburg–Landau theory for a homogeneous, global order parameter in which gauge fields are screened by the Anderson–Higgs mechanism and which usually corresponds to the microscopic BCS pairing mechanism. The type III superconductivity is described by a topological gauge theory and corresponds to an inhomogeneous network of condensate droplets getting connected by tunneling pairs percolation and is destroyed by vortex liberation instead of pairs breaking. The underlying physics is the generalization of the BKT physics to 3D. The corresponding predicted modified exponential VFT scaling of the resistance is fully confirmed by experiment. In 2D, only this second type of superconductivity survives due to strong infrared divergences in the Ginzburg–Landau theory, in 3D both types of superconductivity are possible. There are strong hints that the type of superconductivity that we describe here is associated with the BEC of strongly bound electron pairs and is realized in the high‐*T*_c_ materials.

## 6 Experimental Section

### Preparation of the NbN Film

A RF reactive magnetron sputter was used to deposit a 20‐nm‐thick NbN film on a MgO (100) substrate in an ultrahigh vacuum chamber with the base pressure of 3.9 ×  10^−9^ Torr.

The argon/nitrogen flow rate was fixed to 12:0.5. The fixed gas pressure of 3 mTorr and the fixed RF power of 120 W were used. The argon plasma (12 sccm) struck the NbN target and atoms or molecules were ejected from the target surface. These atoms or molecules travelled toward the silicon substrate with the high temperature of 800 ° C and deposited as the NbN film.

### Preparation of the NbTiN Film

The detailed description regarding the preparation of the disordered nonstoichiometric 86‐nm‐thick NbTiN film is given in ref. [41]. In short, the film was deposited on a Si (100) substrate by the RF reactive magnetron co‐sputtering from two separate NbN (99.5%) and TiN (99.5%) targets at 800 °C. The base pressure in the chamber was less than 9 ×  10^−9^ Torr, and the gas pressure was controlled at 3 mTorr during the deposition. The RF sputtering powers of both targets were set as 100 W. The gas flow rate ratio of argon and nitrogen was 12:0.5. The argon plasma (12 sccm) struck two targets, and atoms or molecules were ejected from the target's surface. These atoms or molecules travelled toward the silicon substrate with the high temperature of 800 °C and were deposited as the NbTiN film. The large lattice mismatch between NbTiN and Si possibly led to disordered and inhomogeneous nature of the NbTiN film.

### Electrical Measurements

The low‐temperature four‐terminal resistance measurements were performed in an Oxford Triton 200 cryo‐free ^3^He/^4^He dilution fridge. A Keithley 2400 current source meter was used to provide a constant dc current that flows from the source to the drain contact. On the other hand, a Keithley 2000 multimeter was used to measure the voltage drop between the two voltage probes.

### Magnetization Measurements

Magnetic susceptibility measurements were carried out using a dc superconducting quantum interference device magnetometer. Both zero‐field‐cooled (ZFC) and field‐cooled (FC) regimes were used.

### Statistical Analysis

Sample size (*n*) for each statistical analysis was *n* = 1. The software used for statistical analysis was Origin.

## Conflict of Interest

The authors declare no conflict of interest.

## Data Availability

The data that support the findings of this study are available from the corresponding author upon reasonable request.

## References

[1] M. Tinkham , Introduction to Superconductivity, Dover Publications, New York 1996.

[2] D. Kowal , Z. Ovadyahu , Solid State Commun. 1994, 90, 783.

[3] T. I. Baturina , A. Yu. Mironov , V. M. Vinokur , M. R. Baklanov , C. Strunk , Phys. Rev. Lett. 2007, 99, 257003. 18233550 10.1103/PhysRevLett.99.257003

[4] M. C. Diamantini , P. Sodano , C. A. Trugenberger , Nucl. Phys. 1996, B474, 641.

[5] V. M. Vinokur , T. I. Baturina , M. V. Fistul , A. Yu. Mironov , M. R. Baklanov , C. Strunk , Nature 2008, 452, 613. 18385735 10.1038/nature06837

[6] M. C. Diamantini , C. A. Trugenberger , V. M. Vinokur , Commun. Phys. 2018, 1, 77.

[7] D. Das , S. Doniach , Phys. Rev. 1999, B 60, 1261.

[8] M. C. Diamantini , A. Yu. Mironov , S. V. Postolova , X. Liu , Z. Hao , D. M. Silevitch , Ya. Kopelevich , P. Kim , C. A. Trugenberger , V. M. Vinokur , Phys. Lett. A 2020, 384, 126570.

[9] M. C. Diamantini , L. Gammaitoni , C. A. Trugenberger , V. M. Vinokur , J. Supercond. Novel Magn. 2019, 32, 47.

[10] M. C. Diamantini , C. A. Trugenberger , V. M. Vinokur , in Topological Phase Transitions and New Developments, World Scientific, Singapore 2022, p. 197.

[11] C. A. Trugenberger , Superinsulators, Bose Metals and High‐*T* _ *c* _ Superconductors: The Quantum Physics of Emergent Magnetic Monopoles, World Scientific, Singapore 2022.

[12] B. Sacépé , T. I. Baturina , V. M. Vinokur , M. R. Baklanov , M. Sanquer , Phys. Rev. Lett. 2008, 101, 157006. 18999631 10.1103/PhysRevLett.101.157006

[13] B. Sacépé , T. Dubouchet , C. Chapelier , M. Sanquer , M. Ovadia , D. Shahar , M. Feigel'man , L. Ioffe , Nat. Phys. 2011, 7, 239.

[14] A. M. Polyakov , Phys. Lett. 1975, 59, 82.

[15] R. Jackiw , S. Templeton , Phys. Rev. D 1981, 23, 2291.

[16] A. Glatz , I. S. Aranson , T. I. Baturina , N. M. Chtchelkatchev , V. M. Vinokur , Phys. Rev. B 2011, 84, 024508.

[17] M. C. Diamantini , C. A. Trugenberger , V. M. Vinokur , J. High Energy Phys. 2022, 10, 100.

[18] C. Parra , F. Niemstemski , A. W. Contryman , P. Giraldo‐Gallo , T. H. Geballe , I. R. Fisher , H. C. Manoharan , Proc. Natl. Acad. Sci. U. S. A. 2021, 118, e2017810118. 33846248 10.1073/pnas.2017810118 PMC8072246

[19] D. Mihailovic , V. V. Kabanov , K. A. Müller , Europhys. Lett. 2002, 57, 254.

[20] D. Pelc , M. Vuckovic , M. S. Grbic , Yu. G. Pozek , T. Sasagawa , M. Greven , N. Barisić , Nat. Comm. 2018, 9, 4327. 10.1038/s41467-018-06707-y PMC6193991 30337539

[21] K. M. Bastiaans , D. Chatzopoulos , J.‐F. Ge , D. Cho , W. O. Tromp , J. M. van Ruitenbeek , M. H. Fischer , P. J. de Visser , D. J. Thoen , E. F. C. Driessen , T. M. Klapwijk , M. P. Allan , Science 2021, 374, 608. 34709897 10.1126/science.abe3987

[22] P. Zhou , L. Chen , Y. Liu , I. Sochnikov , A. T. Bollinger , M.‐G. Han , Y. Thu , I. Bozovic , D. Natelson , Nature 2019, 572, 493. 31435059 10.1038/s41586-019-1486-7

[23] A. T. Bollinger , G. Dubuis , J. Yoon , D. Pavuna , J. Misewich , I. Bozovic , Nature 2011, 472, 458. 21525929 10.1038/nature09998

[24] Z. H. Luo , W. Pang , B. Liu , Y.‐Y. Li , B. A. Malomed , Front. Phys. 2021, 16, 32201.

[25] F. Wilczek , Phys. Rev. Lett. 1992, 69, 132. 10046207 10.1103/PhysRevLett.69.132

[26] A. P. Nielsen , A. B. Cawthorne , P. Barbara , F. C. Wellstood , C. J. Lobb , R. S. Newrock , M. G. Forrester , Phys. Rev. B 2000, 62, 14380.

[27] M. C. Diamantini , P. Sodano , C. A. Trugenberger , Eur. Phys. J. 2016, B 53, 19.

[28] M. C. Diamantini , C. A. Trugenberger , Nucl. Phys. 2015, 891, 401.

[29] P. Minnhagen , Rev. Mod. Phys. 1987, 59, 1001.

[30] M. C. Diamantini , L. Gammaitoni , C. A. Trugenberger , V. M. Vinokur , Sci. Rep. 2018, 8, 15718. 30356062 10.1038/s41598-018-33765-5 PMC6200790

[31] M. Ovadia , D. Kalok , I. Tamir , S. Mitra , B. Sacepé , D. Shahar , Sci. Rep. 2015, 5, 13503. 26310437 10.1038/srep13503 PMC4550897

[32] M. G. Vasin , V. N. Ryzhov , V. M. Vinokur , arXiv:1712.00757 , 2017.

[33] S. Sankar , V. M. Vinokur , V. Tripathi , Phys. Rev. B 2018, 97, 020507(R).

[34] M. C. Diamantini , C. A. Trugenberger , V. M. Vinokur , Commun. Phys. 2021, 4, 25.

[35] Y. Aharonov , D. Bohm , Phys. Rev. 1961, 115, 485.

[36] Y. Aharonov , A. Casher , Phys. Rev. Lett. 1984, 53, 319.

[37] L. H. Kaufmann , Formal Knot Theory, Princeton University Press, Princeton 1983.

[38] S. Deser , R. Jackiw , S. Templeton , Phys. Rev. Lett. 1982, 48, 975.

[39] T. J. Allen , M. J. Bowick , A. Lahiri , Mod. Phys. Lett. A 1991, 6, 559.

[40] M. Bergeron , G. W. Semenoff , R. J. Szabo , Nucl. Phys. B 1995, 437, 695.

[41] S.‐Z. Chen , J.‐W. Yang , T.‐Y. Peng , Y.‐C. Chu , C.‐C. Yeh , I.‐F. Hu , S. Mhatre , Y.‐J. Lu , C.‐T. Liang , Supercond. Sci. Technol. 2022, 35, 064003.

[42] F. H. Chen , M. F. Tai , W. C. Horng , T. Y. Tseng , Phys. Rev. B 1993, 48, 1258. 10.1103/physrevb.48.1258 10007990

